# Recent Advances in Indocyanine Green-Based Probes for Second Near-Infrared Fluorescence Imaging and Therapy

**DOI:** 10.34133/research.0583

**Published:** 2025-01-17

**Authors:** Dehong Hu, Menglei Zha, Hairong Zheng, Duyang Gao, Zonghai Sheng

**Affiliations:** ^1^ Research Center for Advanced Detection Materials and Medical Imaging Devices, Institute of Biomedical and Health Engineering, Shenzhen Institute of Advanced Technology, Chinese Academy of Sciences, Shenzhen 518055, P. R. China.; ^2^ State Key Laboratory of Biomedical lmaging Science and System, Shenzhen 518055, P. R. China.; ^3^ Dongguan Key Laboratory of Chronic Inflammatory Diseases, the First Dongguan Affiliated Hospital, Guangdong Medical University, Dongguan 523710, P. R. China.

## Abstract

Fluorescence imaging, a highly sensitive molecular imaging modality, is being increasingly integrated into clinical practice. Imaging within the second near-infrared biological window (NIR-II; 1,000 to 1,700 nm), also referred to as shortwave infrared, has received substantial attention because of its markedly reduced autofluorescence, deeper tissue penetration, and enhanced spatiotemporal resolution as compared to traditional near-infrared (NIR) imaging. Indocyanine green (ICG), a US Food and Drug Administration-approved NIR fluorophore, has long been used in clinical applications, including blood vessel angiography, vascular perfusion monitoring, and tumor detection. Recent advancements in NIR-II imaging technology have revitalized interest in ICG, revealing its extended tail fluorescence beyond 1,000 nm and reaffirming its potential as a clinically translatable NIR-II fluorophore for in vivo imaging and theranostic applications for diagnosing various diseases. This review emphasizes the notable advances in the use of ICG and its derivatives for NIR-II imaging and image-guided therapy from both fundamental and clinical perspectives. We also provide a concise conclusion and discuss the challenges and future opportunities with NIR-II imaging using clinically approved fluorophores.

## Introduction

**Fig. 1.**
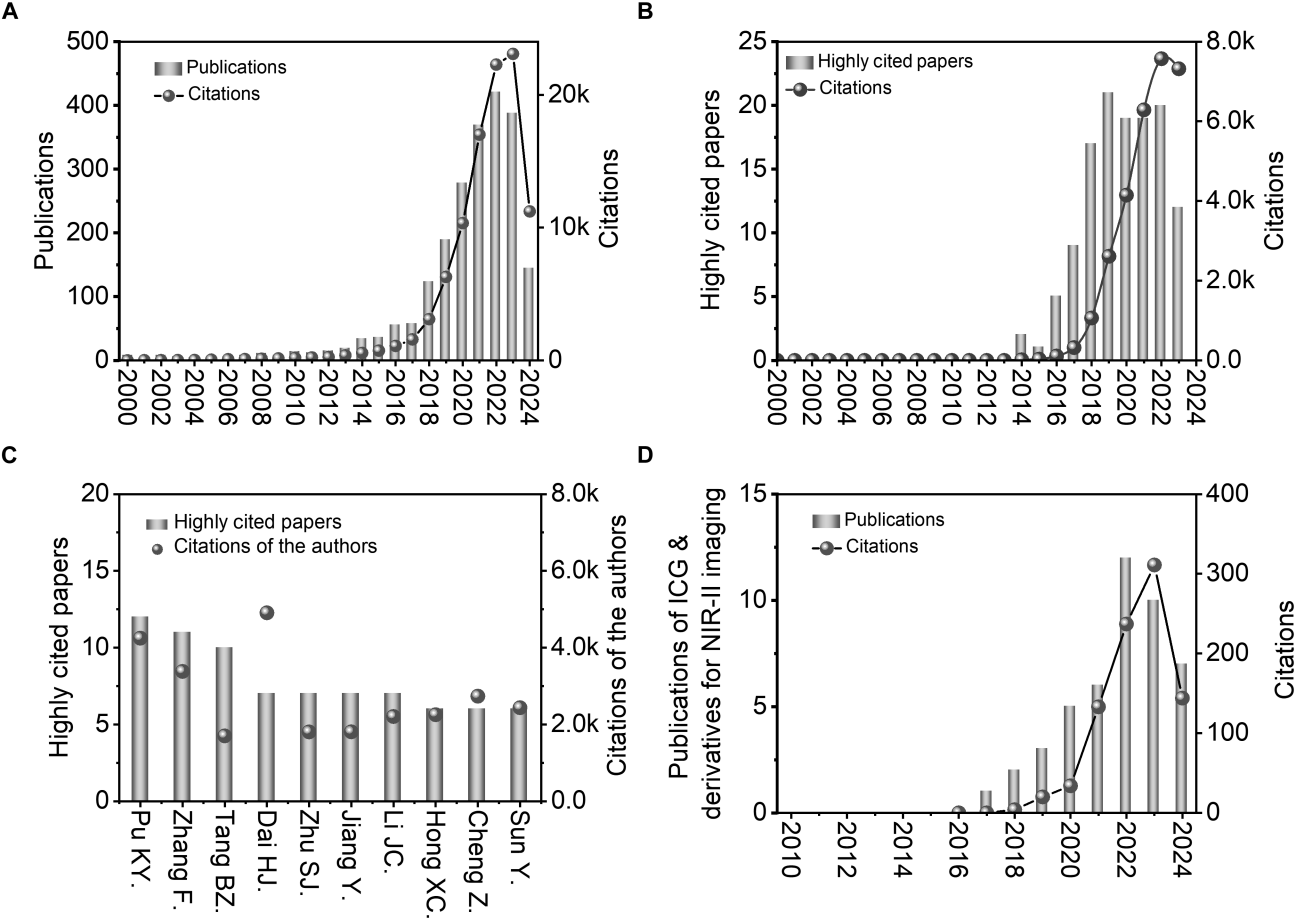
A bibliometric analysis of research publications on NIR-II imaging agents and ICG and its derivatives as NIR-II fluorophores. (A) Annual publications and citations of articles reporting NIR-II imaging agents from 2000 to 2024. (B) Annual number of highly cited papers and their percentage among annual publications on NIR-II imaging agents. (C) Total number of highly cited papers and corresponding citations from the most prolific authors studying NIR-II imaging. (D) Annual publications and citations of articles reporting ICG and its derivatives for NIR-II imaging from 2010 to 2024.

Fluorescence imaging plays an important role in basic laboratory studies and clinical practice because of its nonradiative nature, high sensitivity, and high spatiotemporal resolution [1–3]. Compared to traditional visible [4] and near-infrared (NIR; 700 to 900 nm) imaging [5], second near-infrared (NIR-II; 1,000 to 1,700 nm) fluorescence imaging offers considerably enhanced resolution in deep tissue imaging, which is attributed to its low tissue scattering, reduced tissue absorption, and minimal autofluorescence [6–8]. To date, various kinds of NIR-II fluorescent probes have been developed [9–12], including carbon-based materials [13] (nanotubes [14] and quantum dots [15]), inorganic quantum dots [16], rare earth-doped nanoprobes [17], conjugated polymer particles [18], gold nanoclusters [19], and small-molecule dyes [20,21]. A bibliometric analysis of NIR-II fluorescence imaging-related publications from 2000 and 2024 (Fig. 1) revealed that over 2,260 papers have been published in this field. Additionally, more than 120 highly cited papers have been published, with a marked increase in the number of such papers since 2018. Notably, 80% of these highly cited works have been contributed by Chinese scientists. However, despite the substantial advancements in NIR-II fluorescence imaging, the progress of clinically applicable fluorescent probes in this biological window remains limited.

ICG, a prominent NIR fluorescent probe, was approved by the US Food and Drug Administration (FDA) in 1959 for medical diagnosis. It is broadly applied in cardiac output measurement [22], liver function assessment [23,24], vascular imaging [25], and other fields [26,27] because of its excellent biocompatibility, lack of liver and kidney toxicity, nondeposition in the skin, nonparticipation in biological transformation within the body, and its ability to be mainly metabolized through the liver-intestinal circulation and excreted from the body [28,29]. In 2018, Carr et al. [30] measured the fluorescence spectrum of ICG in the 700- to 1,600-nm wavelength range and found that it exhibits strong tail emission in the NIR-II biological window. This discovery prompted extensive research on the application of ICG for NIR-II fluorescence imaging in clinical and preclinical studies [31–35]. Despite achieving substantial improvements in NIR-II fluorescence imaging, the following challenges remain to be resolved: (a) inherent instability of ICG molecules under light and heat treatment, (b) short in vivo half-life of ICG (3 to 5 min), and (c) no molecular targeting ability [36,37]. To date, various ICG-based probes, including ICG derivatives, ICG-targeted molecular probes, and ICG-loaded nanocomplexes, have been developed to address these limitations. These probes have been applied in intraoperative diagnosis, endoscopic imaging, imaging-guided surgery, phototherapy, and combination therapy with drugs [38–40].

**Fig. 2.**
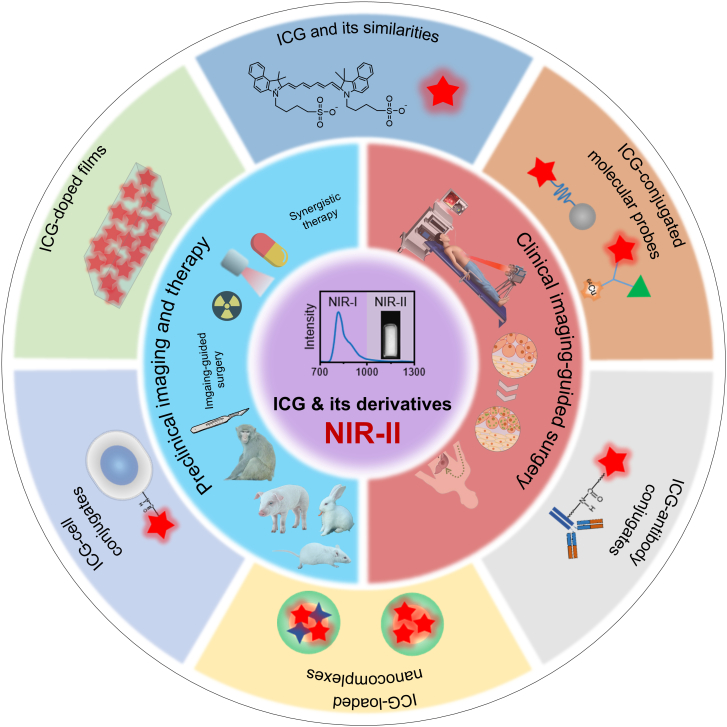
Schematic illustration of ICG-based probes for NIR-II fluorescence imaging and image-guided therapy in preclinical and clinical settings.

In this review, we introduce the types and optical properties of ICG-based probes, discuss their fluorescence imaging in the NIR-II range and image-guided therapy in preclinical and clinical studies, and provide an outlook on the opportunities and challenges related to ICG-based NIR-II fluorescence imaging in the future (Fig. 2).

## Classification of ICG-Based Probes

**Fig. 3.**
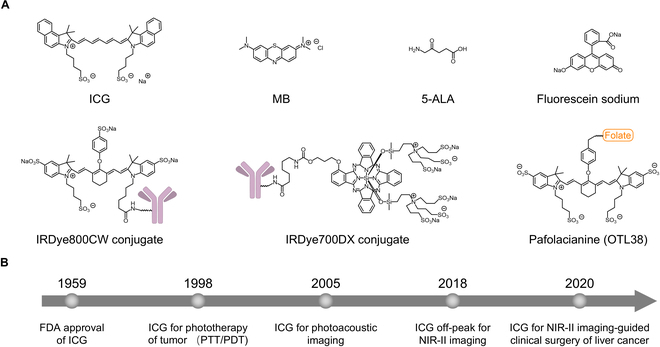
(A) Chemical structures of currently FDA-approved fluorescent molecular imaging probes. (B) Timeline of ICG for different applications after approval by the FDA.

Organic dyes have a high potential for use as molecular imaging probes in clinical application as compared to inorganic fluorophores since their clear structure, good pharmacokinetics, and superior biocompatibility [10,41–44]. Several types of small-molecular organic dyes and their conjugates have been utilized clinically, including ICG, methylene blue (MB), 5-aminolevulinic acid (5-ALA), fluorescein sodium, IRDye800CW conjugate, IRDye700CW conjugate, and pafolacianine (OTL38) [45–47] (Fig. 3A). Among them, ICG is the most prominent NIR fluorescent probe and is used more than half a century in clinics (Fig. 3B).

As a typical symmetric benzothiazole cyanine dye, ICG contains 2 nitrogen-containing heterocycles as terminal groups connected by a polymethine linker. Its structure features hydrophobic characteristics, yet the presence of 2 sulfonate groups enhances its water solubility. The absorption and fluorescence emission spectra of ICG are influenced by its concentration and the solvent used. In solvents such as ethanol and dimethyl sulfoxide, ICG exists predominantly in a monomeric form; however, at high concentrations in water, it tends to form H-aggregates [48,49]. This characteristic, along with the broad fluorescence spectrum and considerable overlap between the absorption and emission spectra, results in a pronounced aggregation-induced fluorescence quenching effect [50,51]. Despite this, ICG has a high fluorescence quantum yield and molar extinction coefficient (ɛ = 121,000 M^−1^ cm^−1^), enabling it to produce bright fluorescence even at low concentrations. To address its limitations in various applications, researchers have developed a range of ICG-based probes [28,52]. Here, we provide a preliminary comparison of their properties, advantages, and disadvantages (Table 1).

**Table 1.** Comparison of different types of ICG-based probes

Category	Description	Properties	Advantages	Disadvantages
ICG derivatives	Modify the structure of ICG via molecular engineering	Long emission wavelength, high quantum yield, good solubility, and other properties	Good optical performance, relatively simple structure, and great potential for clinical translation	Complex synthesis steps, time-consuming
ICG-conjugated molecular probes	Coupling of small functional molecules	Multimodal imaging ability and responsive imaging ability	Respond to specific stimuli and have great potential for multimodal imaging	Limited targeting performance, with sensitivity needing further improvement
ICG–antibody conjugates	Conjugated with targeting molecules such as peptides and antibodies	Active-targeting imaging ability	Precisely target diseased cells, improving diagnostic accuracy and therapeutic monitoring capabilities	Antibody modification is complex, cost is relatively high, and large-scale production presents challenges
ICG–nanocarrier complexes	ICG encapsulated within the nanocarriers including liposome, albumin, and others	Improving stability, enhancing brightness, extending the blood half-life	High stability, tunable targeting ability, and great potential for multifunctionalization	The in vivo safety of nanocarriers is complex, making clinical translation challenging

### ICG similarities

**Fig. 4.**
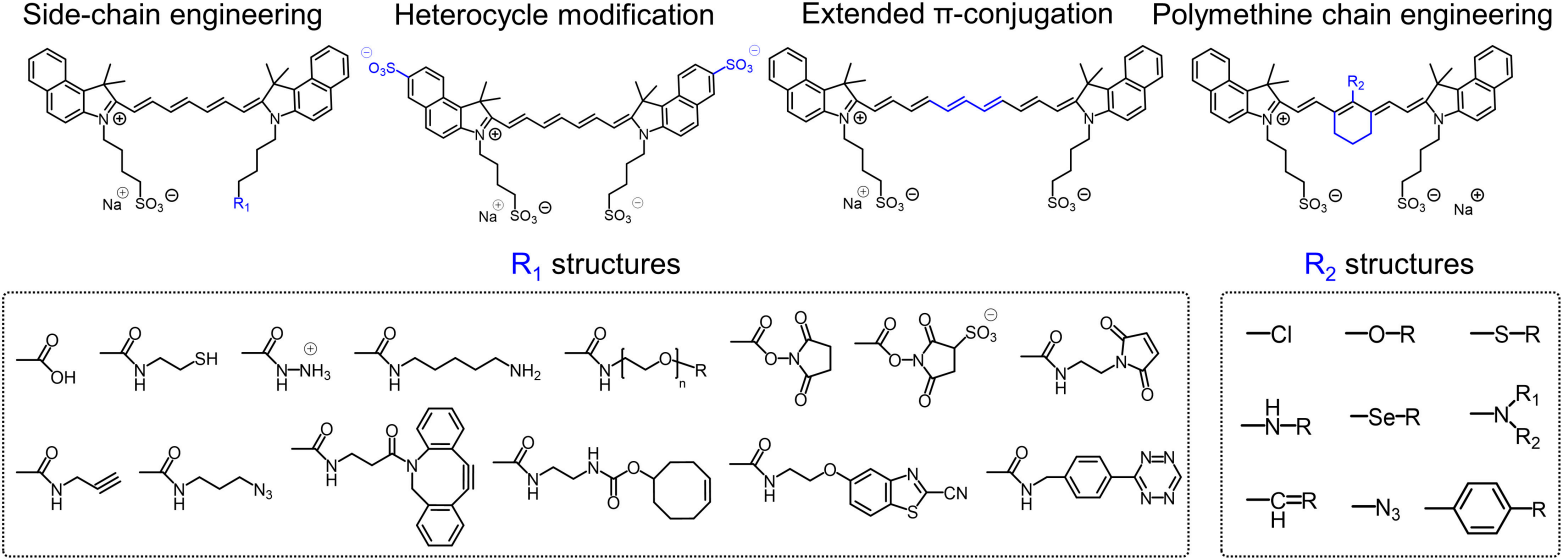
Molecular engineering of ICG to fabricate its similarities.

Researchers have developed a series of ICG derivatives by introducing various substituents onto the molecular framework of ICG, allowing for the modulation of its optical and physicochemical properties based on its molecular structure [53,54]. Depending on the position of the substituents, these derivatives can be categorized into 4 main types (Fig. 4): (a) Side-chain engineering: By introducing functional groups such as carboxyl, thiol, and azide onto the side chains, the modifiability of the molecule is enhanced. (b) Heterocycle modification: Adding highly polar groups can improve water solubility while also enhancing biocompatibility. (c) Extended π-conjugation: The polymethine chain is a key structural element determining the spectral characteristics of ICG. By extending the π-conjugation, both the emission and absorption wavelengths can be red-shifted. (d) Polymethine chain engineering: Although extending the π-conjugation leads to red shifts in wavelength, it can also reduce quantum yield and molecular stability. Modifying the polymethine backbone offers a strategic approach to enhance the fluorescence brightness of long-wavelength ICG analogs [55].

### ICG-conjugated molecular probes

ICG-conjugated molecular probes refer to ICG molecules that are linked to functional small molecules to enhance their imaging capabilities, thereby providing additional information for disease diagnosis. These probes can be primarily categorized into 2 types: (a) Stimuli-responsive bioimaging: By coupling fluorescent quenching molecules with stimuli-responsive linkers, activation imaging can be achieved in physiological environments where specific stimuli are present. This approach not only captures information about the stimuli associated with disease progression but also effectively enhances the signal-to-noise ratio (SNR) in imaging [56–58]. (b) Active-targeting multimodal imaging: By conjugating radionuclides and targeting small molecules, these probes enable active-targeting multimodal imaging. This strategy leverages the advantages of different imaging modalities to improve the accuracy of disease diagnosis and provides multidimensional molecular information, serving as a valuable tool for the in-depth investigation of disease mechanisms [59,60].

### ICG–antibody conjugates

ICG–antibody conjugates are compounds formed by chemically linking ICG to antibodies via covalent bonds. Antibodies possess high specificity and targeting capabilities, enabling them to recognize and bind precisely to specific antigens, often including disease-related biomarkers such as proteins on tumor cell surfaces. By conjugating ICG with antibodies, these conjugates leverage the fluorescence imaging properties of ICG alongside the targeting ability of antibodies, facilitating accurate localization and visualization of disease-associated cells or tissues [61–63]. Furthermore, the preparation and purification of ICG–antibody conjugates are relatively simple, offering promising potential for clinical translation [63–65]. Several products are currently undergoing clinical trials.

### ICG–nanocarrier complexes

ICG–nanocarrier complexes refer to compounds formed by loading ICG onto various nanocarriers through methods such as physical adsorption, encapsulation, or covalent bonding [66,67]. These complexes typically offer the following advantages: (a) Enhanced stability: Nanocarriers can encapsulate or adsorb ICG, protecting it from external environments and improving its stability [68,69]. (b) Improved targeting ability: Nanocarriers can be functionalized with targeting ligands, such as antibodies, peptides, or small molecules, allowing for active targeting of specific cells or tissues. Additionally, their size enables them to exploit the enhanced permeability and retention (EPR) effect in tumor tissues for passive targeting [70,71]. (c) Multifunctional integration: In addition to carrying ICG, nanocarriers can load other drugs or functional molecules, facilitating the integration of imaging, therapy, and other functionalities [72,73]. (d) Extended imaging window: By tuning the size and surface properties of the nanocarriers, the circulation time of ICG in the bloodstream can be prolonged, extending the duration of imaging [74–76].

## Preclinical Applications of ICG-Based Probes

In this section, we describe the utilization of ICG-based probes for fluorescence imaging in the NIR-II window and therapeutic guidance. We sequentially introduce ICG and its similarities, ICG-conjugated molecular probes, ICG–antibody conjugates, ICG–nanocarrier complexes, and other hybrids for preclinical imaging, diagnosis, and theranostics of various diseases.

### ICG and its similarities for NIR-II fluorescence imaging

ICG and its analogs for NIR-II fluorescence imaging can be categorized into 2 types based on their emission wavelength differences: (a) those similar to ICG, which exhibit tail emission in the NIR-II range; (b) those with a maximum emission peak in the NIR-II range. We have preliminarily summarized the structures and properties of some representative molecules (Table 2).

**Table 2.** Chemical structures and optical properties of ICG and its analogs

	Fluorophore	Structure	Absorption wavelength/emission wavelength	Water-soluble	Reference
Tail emission in the NIR-II region	ICG	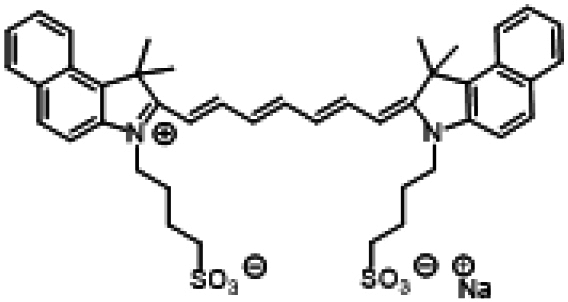	780/810	Yes	[154]
	IRDye800CW	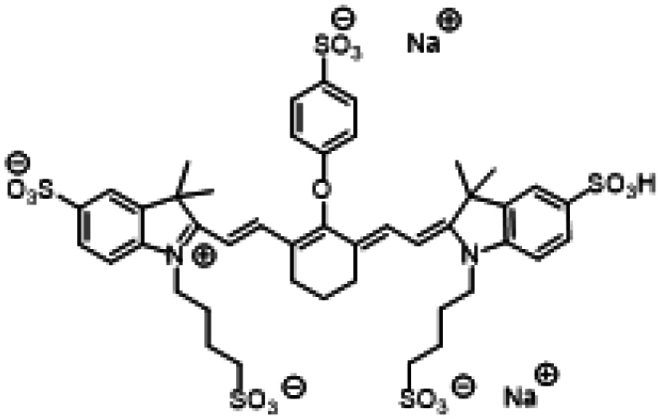	774/789	Yes	[155]
	IR-783	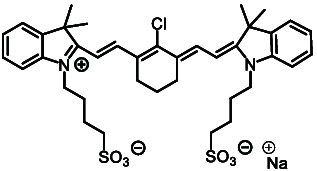	793/858	No	[156]
	IR12-N3	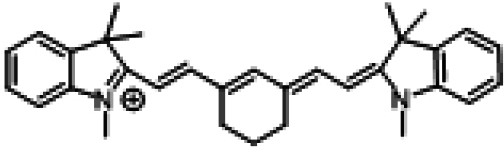	770/800		[157]
Maximum emission peak located in the NIR-II region	IR-1048	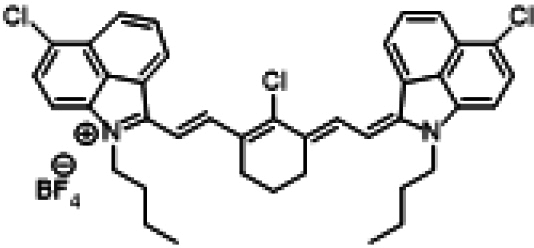	980/1,046	No	[158]
	IR-1061	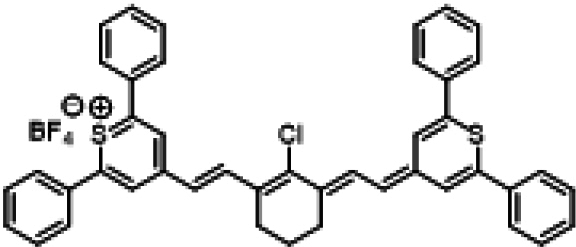	1,074/1,132	No	[158]
	IR-26	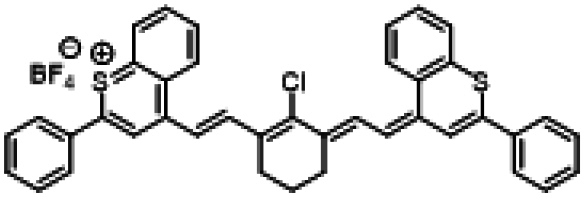	1,073/1,138	No	[159]
	FD-1080	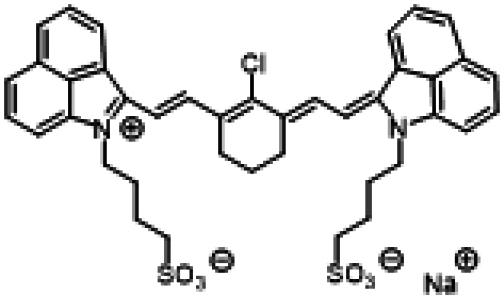	1,046/1,080	Yes	[85]
	Flav7	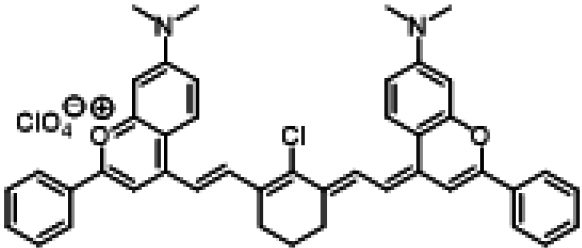	1,026/1,045	No	[160]
	LZ-1105	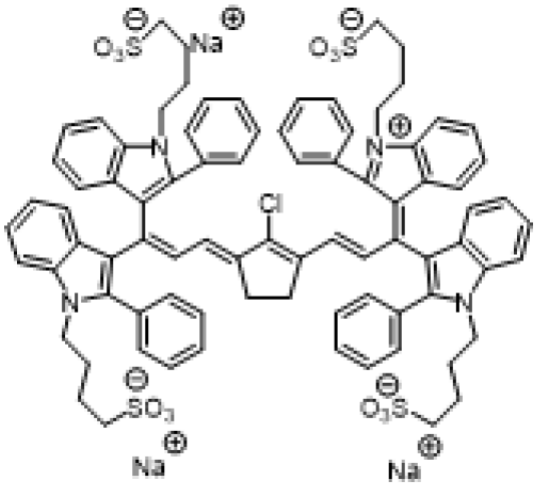	1,041/1,105	Yes	[161]
	CX-3	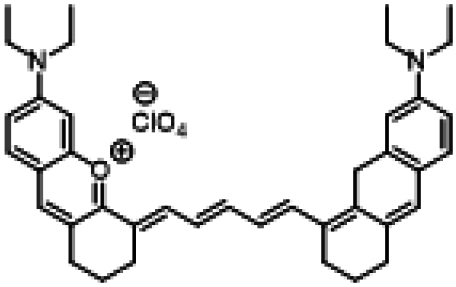	1,089/1,140	No	[162]
	BTC-1070	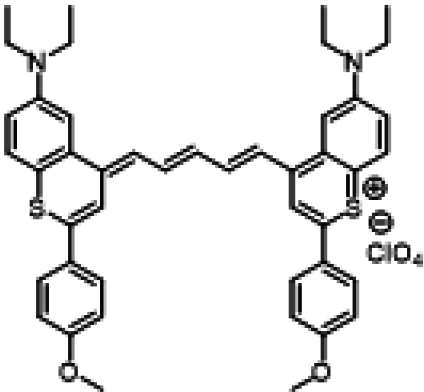	1,014/1,070	No	[163]
	5H5-1069	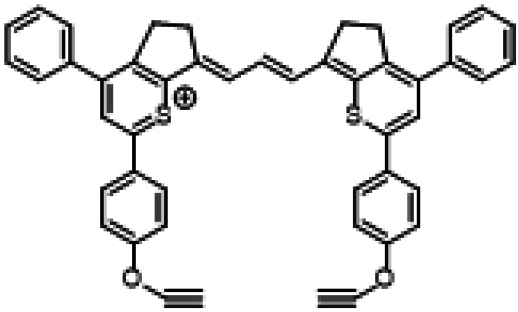	1,069/1,125	No	[164]

**Fig. 5.**
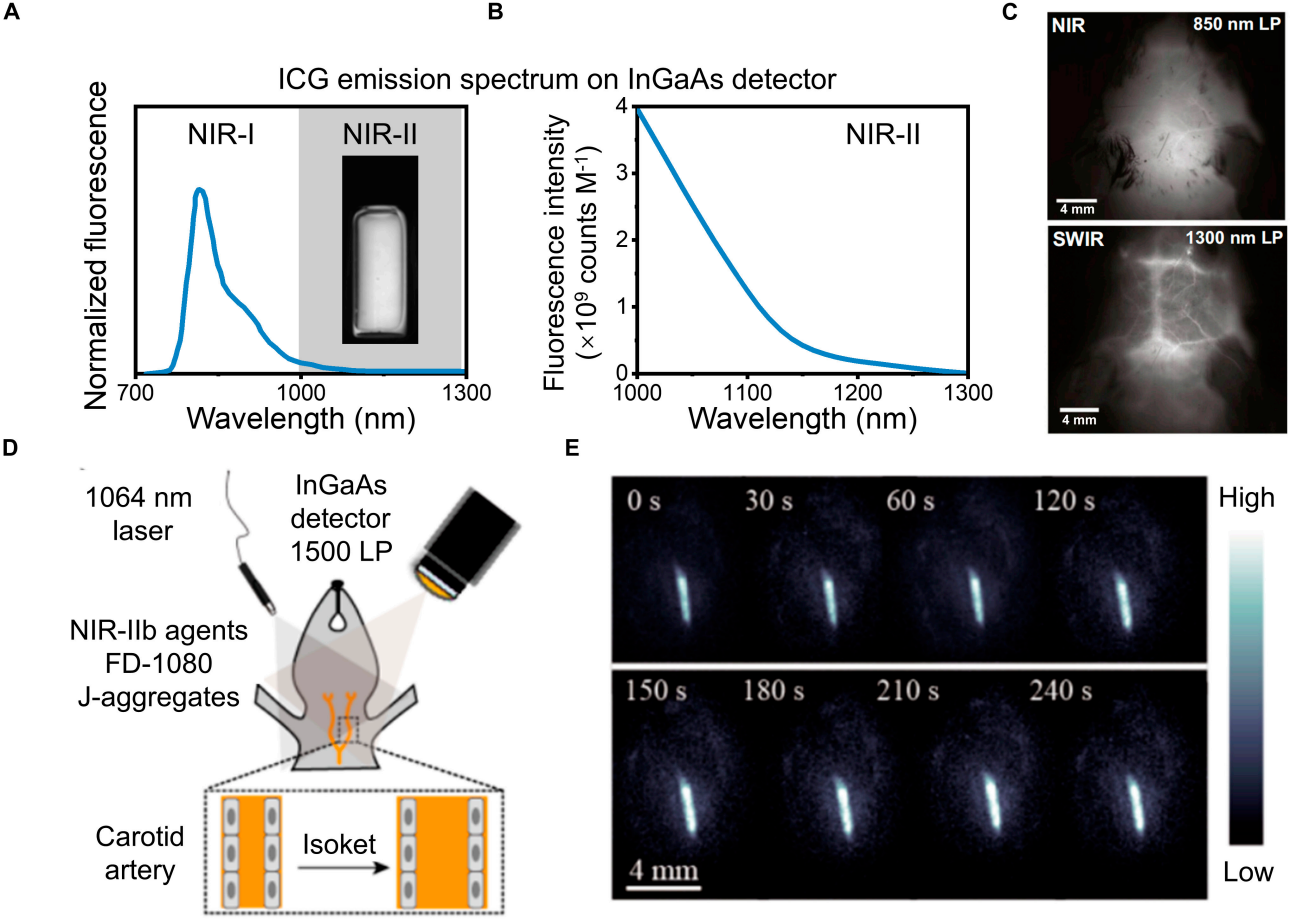
ICG for NIR-II fluorescence imaging. (A) Full emission spectrum of ICG and the NIR-II photograph of the ICG solution. The InGaAs detector can recover the true emission tail of ICG. (B) Fluorescence signal of ICG in the NIR-II region. (C) NIR-I (up) and NIR-II (down) brain vascular fluorescence imaging using ICG and different long-pass filters. Reprinted from [30] with permission from the National Academy of Sciences. (D) Scheme of the hypotension process and (E) dynamic bioimaging of carotid artery beyond 1,500-nm window after Isoket administration. Reprinted from [87] with permission from the American Chemical Society.

Several NIR-I cyanine dyes, including ICG, IR12-N3, and IR-783, have demonstrated NIR-II tail emissions for bioimaging applications [77,78]. Among these, ICG is the most widely utilized, with its tail emission first detected using an InGaAs detector by Carr’s group [30] (Fig. 5A to C). Preclinical NIR-II imaging with ICG has expanded from small animals to large animals, achieving spatial resolutions across macroscopic, mesoscopic, and microscopic scales. Carr et al. [30] visualized brain vasculature and hindlimb vessels in mice using 0.2 mg/kg ICG, achieving a vessel full width at half maximum (FWHM) of 210 μm. Li and colleagues [79] achieved high-resolution cerebrovascular imaging (10.7 μm) with a penetration depth of 850 μm using wide-field microscopy, further enhancing resolution to 2.66 μm with confocal microscopy. Cai and colleagues [80] imaged rat bile ducts, achieving a resolution of 455.5 μm and a signal-to-background ratio (SBR) of 5.22. In pigs, Davis and colleagues [81] demonstrated improved spatial and contrast resolution in brain angiography compared to NIR-I imaging, while Wu et al. [82] visualized microvascular networks during flap perfusion surgery, with an FWHM of 6.6 mm and an SBR of 2.11. Qian and colleagues [83] used rhesus macaques to achieve cerebral vessel imaging with confocal microscopy, attaining high spatial (~8 μm) and temporal (25 frames per second) resolution, an SBR of 50, and a penetration depth of 470 μm.

In addition to dyes with tail emissions in the NIR-II region, scientists have developed molecules with emission wavelengths directly in the NIR-II range by modifying ICG’s structure. Zhang’s team introduced FD-1080 as a notable example, extending its absorption and emission peaks into the NIR-II region (1,046/1,080 nm) through polymethine chain engineering [84]. The quantum yield increased dramatically from 0.31% to 5.94% after combination with fetal bovine serum (FBS). This complex demonstrated superior tissue penetration depth and spatial resolution when excited at 1,064 nm. Imaging applications included high-resolution visualization of mouse hindlimb and brain vasculature and dynamic monitoring of breathing in both awake and anesthetized mice [85]. Furthermore, in the presence of 1,2-dimyristoyl-sn-glycero-3-phosphocholine (DMPC), FD-1080 cyanine dyes self-assembled to form J-aggregates and exhibited absorption and emission at 1,360 and 1,370 nm, respectively [86]. Using this system, the authors demonstrated the advantages of long-wavelength, high-resolution imaging by dynamically monitoring carotid artery changes in hypertensive rats following administration of the clinical vasodilator Isoket (Fig. 5D and E) [87].

ICG and its analogs, whether through tail emissions or peak emissions in the NIR-II region, have shown notable advancements in NIR-II fluorescence imaging. These achievements highlight their versatility in biological and clinical applications and their potential to propel advancements in high-resolution imaging technologies.

### ICG-conjugated molecular probes for NIR-II imaging

**Fig. 6.**
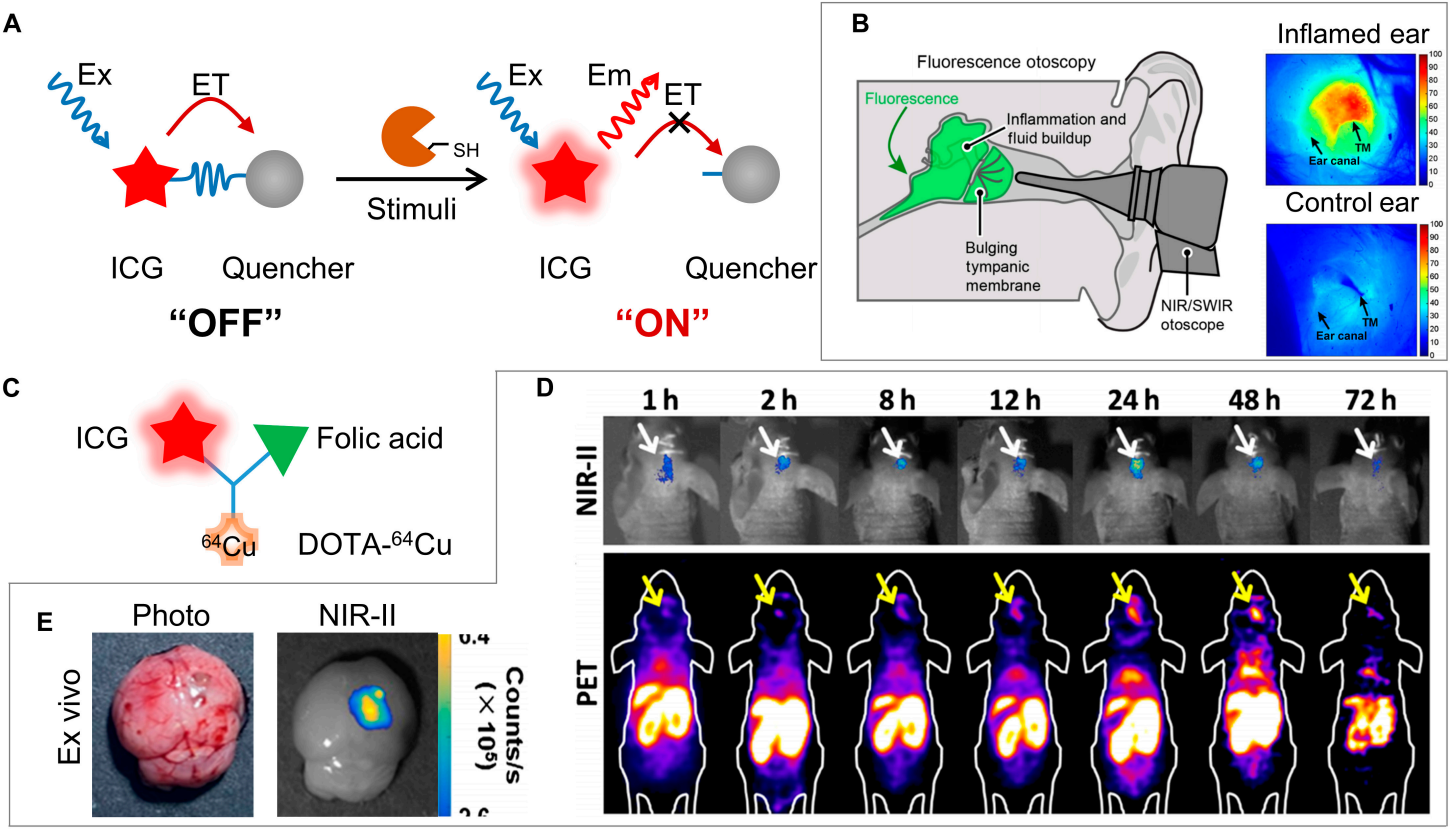
Schemes and applications of ICG-conjugated molecular probes. (A) Designing mechanism of ICG-conjugated protease-cleavable biosensor. (B) In vivo NIR-II otomicroscopy imaging of ear inflammation. Reprinted from [91] with permission from ACS Publications. (C) Molecular design of ^64^Cu-DOTA-FA-ICG nanoprobe. (D) In vivo NIR-II and PET imaging at different time points after ^64^Cu-DOTA-FA-ICG injection. (E) Ex vivo images of the mouse brain at 24 h. Reprinted from [92] with permission from Springer.

Because of its superior optical properties, ICG was conjugated with other functional moieties to prepare molecular probes for in vivo sensing (stimuli-activated fluorescence imaging) and bioimaging. To fabricate ICG-based sensors, ICG was chemically conjugated with an NIR quencher (such as QC-1 or GS-Au25 clusters); thus, the fluorescence of ICG was completely quenched through energy transfer (Fig. 6A). In the presence of external stimuli [88] [such as protease, pH, light, reactive oxygen species (ROS), and glutathione (GSH)], the linker was cleaved, and the fluorescence of ICG was completely recovered [89,90].

Based on this concept, Yim et al. [91] developed a protease-cleavable 6QC-ICG biosensor to detect inflamed ears in the otitis media mouse model (Fig. 6B). By using a customized NIR-II-based otomicroscope, the authors successfully identified the inflamed ear according to the overexpression of cysteine cathepsin proteases in inflammatory immune cells. A 2-fold enhancement of fluorescence intensity was observed in the inflamed ear with an SBR of 2. Thus, the utilization of different stimuli-responsive linkers for ICG-conjugated probes together with an NIR-II imaging system is a promising concept for various applications of in vivo biosensing.

ICG-conjugated molecular probes can also serve as the NIR-II contrast agent for multimodal molecular imaging (Fig. 6C). Shi et al. [92] reported a dual-modal molecular probe named ^64^Cu–1,4,7,10-tetraazacyclododecane (DOTA)–folic acid (FA)–ICG, which can specifically target glioblastoma cells by binding with the folate receptor for NIR-II fluorescence and positron emission tomography (PET) imaging (Fig. 6D and E). Under the guidance of NIR-II images, the glioblastoma tumor mass was completely resected through surgery. Multimodal probes provide complementary information on lesion areas, which is quite promising for clinical diagnosis.

The combination of ICG, functional groups, and other probes of different imaging modalities [such as PET, magnetic resonance imaging (MRI), computed tomography (CT), ultrasound (US), and photoacoustic imaging] not only integrates the advantages of each moiety but also creates new diversities for NIR-II imaging-based clinical theranostics, including but not limited to presurgical diagnosis, intraoperative surgery guidance, and postsurgical efficacy monitoring.

### ICG–antibody conjugates for NIR-II imaging

Numerous monoclonal antibody drugs are currently in clinical use or undergoing clinical trials. These antibodies are designed to specifically bind to overexpressed biomarkers associated with diseases such as cancer and inflammation, thereby facilitating both diagnosis and therapy [93–95]. The antibody is commonly used as the targeting ligand to deliver drugs and fluorophores to the lesion area, which can enhance the accuracy of diagnosis and therapeutic efficacy while minimizing the side effects of drugs and fluorophores [96–98].

**Fig. 7.**
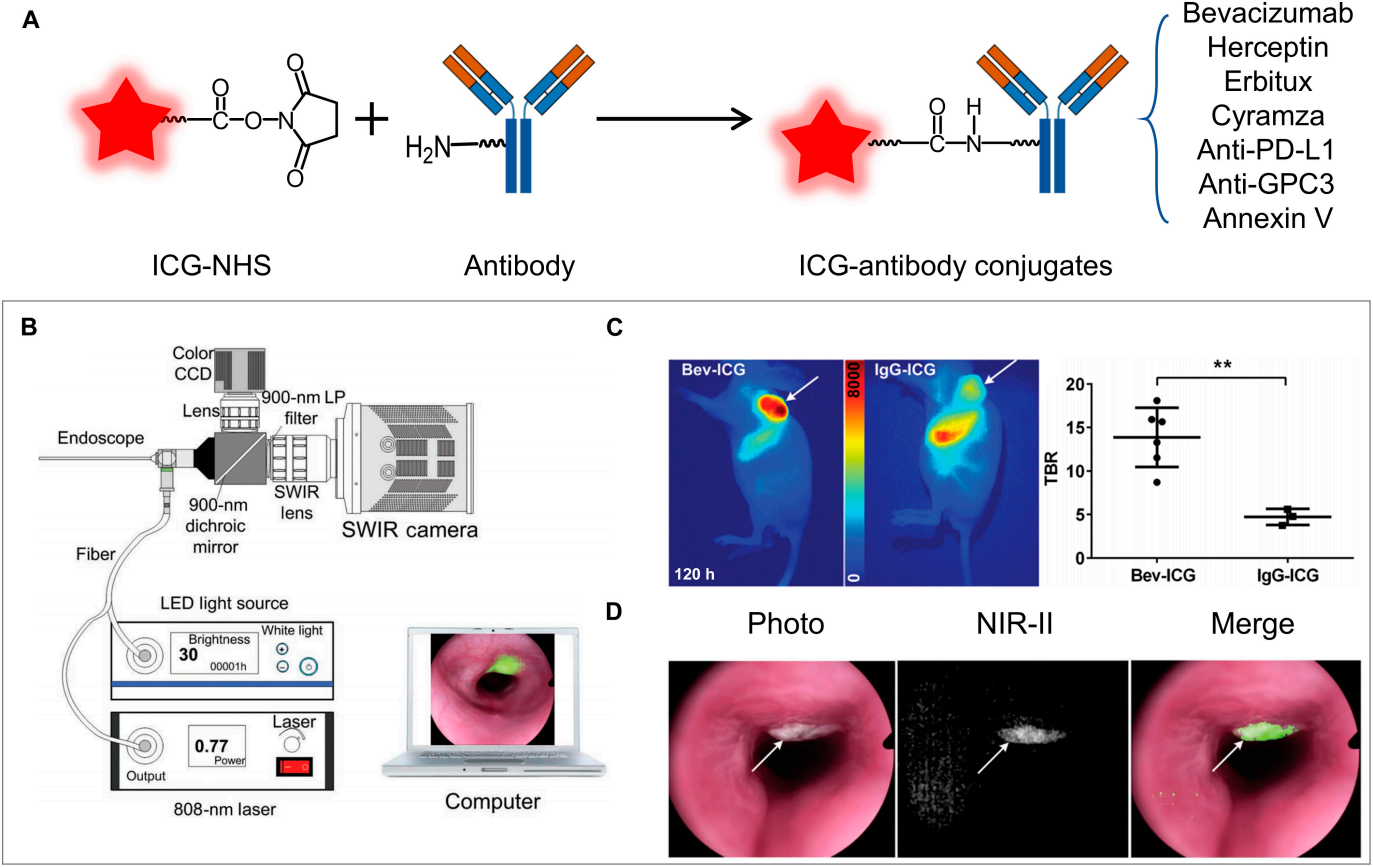
Structure and application of ICG–antibody conjugates. (A) Designing mechanism of ICG–antibody conjugates. (B) Schematic illustration of the NIR-II endoscopy system. Reprinted from [33] with permission from Wiley. (C) NIR-II fluorescence imaging of human colorectal cell 116 (HCT 116) tumor-bearing BALB/c nude mice at 5 d after injection of Bev-ICG (left) compared to the control group injected with IgG-ICG (right) and the SBR of the 2 groups. (D) Simultaneous white-light, NIR-II fluorescence, and merged images of the representative tumor.

Some efforts have also been made to develop ICG–antibody conjugates for tumor-targeted NIR-II fluorescence molecular imaging [61,99]. The antibody was chemically conjugated with ICG by reacting the amine group of the antibody with the amine-activated ester of ICG (Fig. 7A). In 2019, Cheng and colleagues [33] developed an ICG–bevacizumab complex (Bev-ICG) that targeted vascular endothelial growth factor (VEGF) for NIR-II-based endoscopic imaging of colorectal cancer (CRC) (Fig. 7B to D). The Bev-ICG group showed a markedly higher tumor signal, with an SBR of up to 15, as compared to the control group. An NIR-II-based endoscopy system with a subcellular resolution of 20 μm was also designed to work with Bev-ICG in an orthotopic CRC tumor-bearing rat model. Besides bevacizumab, 4 types of ICG–antibody conjugates including herceptin, erbitux, cyramza, and anti-PD-L1 antibody were also designed for diagnosing breast and skin tumors [100]. The NIR-II images displayed 1.5- to 2-fold higher SBR than the NIR-I images. These conjugates were also used to evaluate the efficacy of anticancer drugs. Usually targeting liver cancer poses considerable challenges due to the tendency of most nanoprobes to accumulate in the liver. Recently, Shi et al. [101] successfully achieved specific liver tumor diagnosis using a humanized anti-GPC3 antibody-conjugated ICG probe, which exhibited an SBR of 3. Additionally, a π-conjugation extended ICG derivative (C11) conjugated with Annexin V was developed for NIR-II imaging of tumor apoptosis in a mouse model. This probe achieved a high SBR of 7.2, attributed to its extended emission wavelength [102].

These studies indicate that the integration of ICG–antibody conjugates and advanced NIR-II imaging systems is quite promising for clinical diagnosis and monitoring therapeutic efficacy during the entire disease treatment period.

### ICG–nanocarrier complexes for NIR-II imaging

**Fig. 8.**
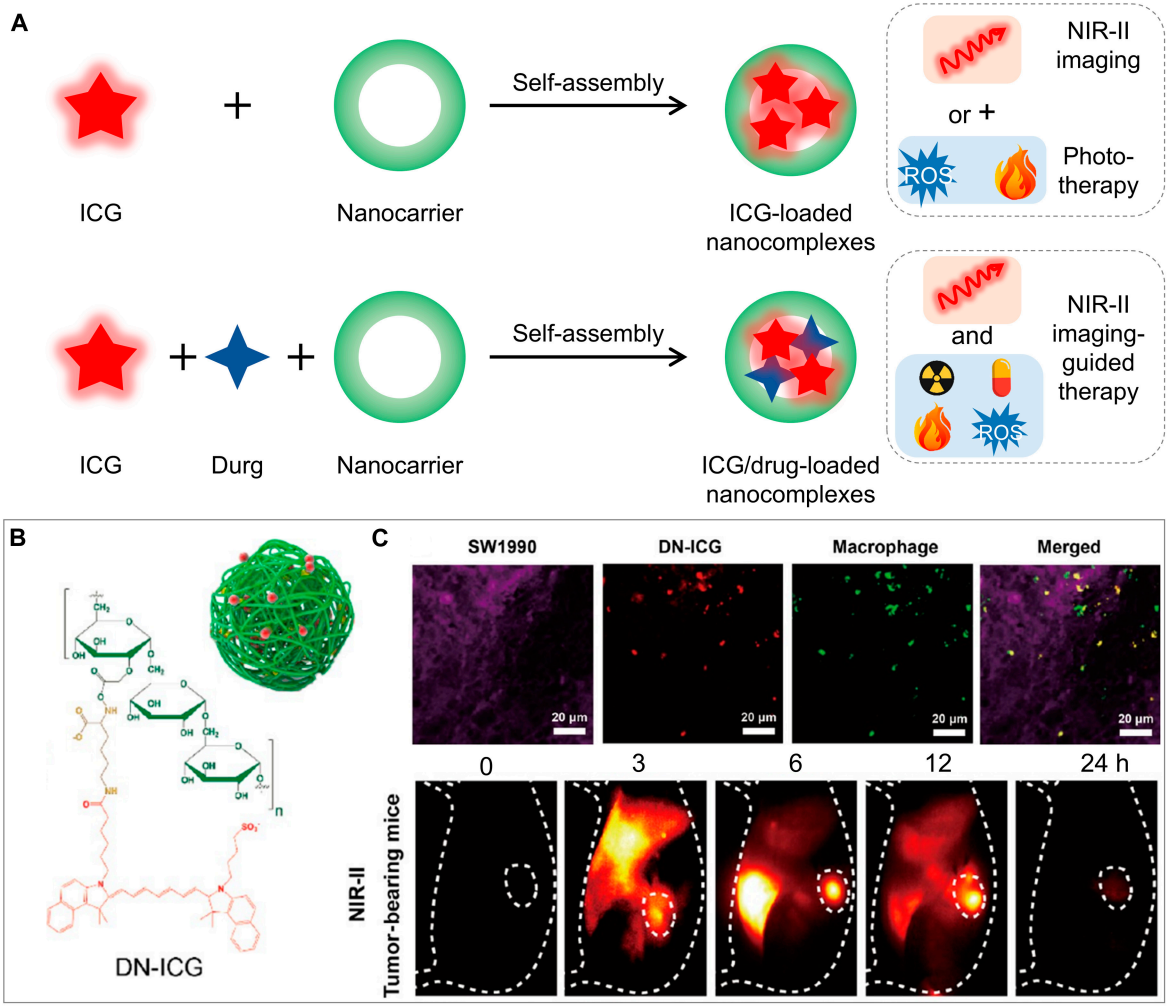
Structure and application of ICG-loaded nanocomplexes for NIR-II imaging. (A) Designing mechanism of ICG or ICG/drug-loaded nanocomplexes for NIR-II imaging and therapy. (B) Chemical structure of the DN-ICG nanoprobe. Reprinted from [119] with permission from ACS Publications. (C) Specific labeling of TAMs with DN-ICG and in vivo NIR-II fluorescence images of DN-ICG in orthotopic pancreatic tumor-bearing mice.

As an amphiphilic molecule, ICG naturally tends to self-accumulate or dock into the hydrophobic pocket of proteins or insert in the lipid bilayer of vesicles through hydrophobic interaction [103,104]. Thus far, many organic/inorganic nanocarriers (such as liposomes [105,106], microbubbles [107], proteins [108], dextran [109], cell membrane fragments [110], and mesopore silica [111]) have been used to encapsulate ICG or ICG/drug to form ICG-loaded nanocomplexes (Fig. 8A) [112–115]. These ICG-loaded nanocomplexes improve optical properties like fluorescence intensity, photostability, and storage stability by preventing structural oxidation and fluorescence quenching in aqueous environments. Consequently, they exhibit superior NIR-II imaging performance compared to free ICG [112–115].

Bhavane et al. [116] encapsulated ICG in PEGylated liposomes to form liposomal ICG for in vivo NIR-II imaging of hindlimb and brain vasculatures. As-prepared liposomal ICG displayed a markedly higher penetration depth and a better SBR of 75 than the SBR of 35 for free ICG. Unlike previous reports, our group proposed a liposome encapsulation strategy to enclose ICG in the hydrophobic pockets of lipids. The NIR-II brightness of the liposome-encapsulated dyes was increased by approximately 40-fold as compared to that with free ICG, thereby facilitating low-dose cerebrovascular imaging. Moreover, the strategy can also be used for other cyanine dyes, including IR780 and FD1080. It enables in vivo dual-color NIR-II fluorescence imaging by tuning the excitation wavelength [117]. To enhance the liposome-ICG system with active-targeting functionality, cell membrane biomimetic liposomes were developed. Leveraging the cell-mimetic properties of biomimetic nanoparticles (NPs) and the superior NIR-II imaging capabilities of ICG, the Neu-NPs synthesized in our study facilitated the visualization of inflamed atherosclerotic plaques in murine and rabbit models, achieving a high SBR [118]. In addition to liposomes, dextran serves as an outstanding carrier for ICG. It not only intensifies the brightness of ICG but also targets tumor-associated macrophages (TAMs) via specific binding to ICAM-3-grabbing nonintegrin-related 1. Utilizing DN-ICG in the NIR-II imaging of a pancreatic cancer-bearing mouse model, we accomplished a high SNR of approximately 7 and enabled a deep tissue penetration of up to 0.5 cm (Fig. 8B and C) [119].

In summary, the development of ICG nanocarriers has expanded its NIR-II imaging potential, and encapsulation strategies have enhanced its optical and imaging properties. Looking forward, future research should focus on optimizing nanocomplex design and functionality, like exploring new combinations for higher imaging sensitivity and specificity. Also, studies on in vivo stability and biocompatibility are needed for clinical translation. Moreover, the use of ICG-loaded nanocarriers for theranostic applications is promising and worth exploring, such as co-delivering drugs for simultaneous diagnosis and treatment. Overall, continued efforts in this field will likely drive substantial advancements in biomedical imaging and therapeutics.

### Other types of ICG derivatives for NIR-II imaging

The applications of ICG derivatives as NIR-II fluorescent probes have extended from nanoscale to micrometer-scale and centimeter-scale. Cell-based theranostic agents for NIR-II fluorescence-guided therapy of metastatic tumors were designed. They conjugated RGD peptides, upconversion NPs (UCNPs), and Rose Bengal (RB) with red blood cells and labeled the cells with ICG, thus endowing NIR-II imaging ability, active targeting, and enhanced photodynamic therapy (PDT) of malignant tumors. In addition to tumor theranostics, ICG is also used for stem cell tracking in vivo. In 2022, Jiang and colleagues [120] reported ICG-labeled adipose-derived stem cell sheets for monitoring the repairing process of urethral mucosa defect in a rabbit model. The ICG–cell conjugates could be successfully tracked in vivo for up to 8 weeks by using NIR-II optical imaging. This tracking method offers a novel option for tissue engineering to track the repair process of stem cells. In addition to ICG–cell conjugates, ICG was also doped in nontoxic chitosan to prepare a centimeter-scale film for sublingual administration. Lu and colleagues [121] prepared 2 types of ICG-doped films with different release rates for noninvasive swallowing evaluation and inflammation detection through NIR-II imaging in mouse models. After evaluation with the NIR-II imaging system, this noninvasive delivery strategy displayed signal enhancement comparable to that of the conventional intravenous injection method. Currently, research on ICG-labeled cell tracking and ICG-based devices for NIR-II imaging-guided applications is limited and still in its early stages. Developing new ICG-based platforms with different spatial scales for various preclinical applications will provide more opportunities for clinical translation.

### ICG-based probes for NIR-II imaging-guided therapy

ICG, as a dopant, can act as a fluorophore and a therapeutic agent by generating heat and ROS, thus making it suitable for imaging and phototherapy [photothermal therapy (PTT)/PDT] in preclinical studies. The use of nanocarriers enhances the delivery of ICG to specific lesions through various mechanisms such as molecular interactions, mechanical forces, or blood vessel permeation. Consequently, ICG-based probes are emerging as promising tools for clinical diagnostics and molecular imaging-guided therapeutic interventions in disease management (Table 3) [122–124].

**Table 3.** Summary of ICG-loaded nanocomplexes for NIR-II imaging-guided therapy

Loaded materials	Nanocarriers	Targeting mechanism	Biomedical applications	Reference
ICG + DOX	SW1990 cell membrane	Homologous targeting	Phototherapy + chemotherapy of pancreatic cancer	[130]
ICG + EGCG + Cu^2+^ + DOX	-	EPR effect	Phototherapy + chemotherapy of 4T1 tumor	[133]
ICG + MTX + CA	-	EPR effect	Phototherapy + chemotherapy of 4T1 tumor	[127]
ICG + MTX	PEG-CH=N-MTX	EPR effect	PTT + chemotherapy of HeLa tumor	[132]
ICG-iRGD	HSA	α_v_β_3_ integrin	Imaging-guided phototherapy of U256 and C6 brain tumors	[165]
ICG	Microbubbles	Focused ultrasound	Imaging BBB opening and enhancing phototherapy of glioma	[107]
ICG + IR-1061	PCL-PEG	-	Imaging of blood vessels and PTT of MCF-7 cells	[126]
ICG	HV-Gd-cRGD	α_v_β_3_ integrin	Imaging-guided surgery and radiosensitization of 4T1 tumor	[135]
ICG	Abraxane@MoSe_2_	EPR effect	Photothermal therapy + chemotherapy of pancreatic cancer	[134]
ICG + UCNPs + RB + RGD	Red blood cell	α_v_β_3_ integrin	NIR-II fluorescence imaging-guided surgery and enhanced PDT of metastatic tumors	[166]
mAb-ICG	Cancer cell	MCT4	NIR-II fluorescence imaging-guided surgery and PTT of U87MG tumor	[167]

**Fig. 9.**
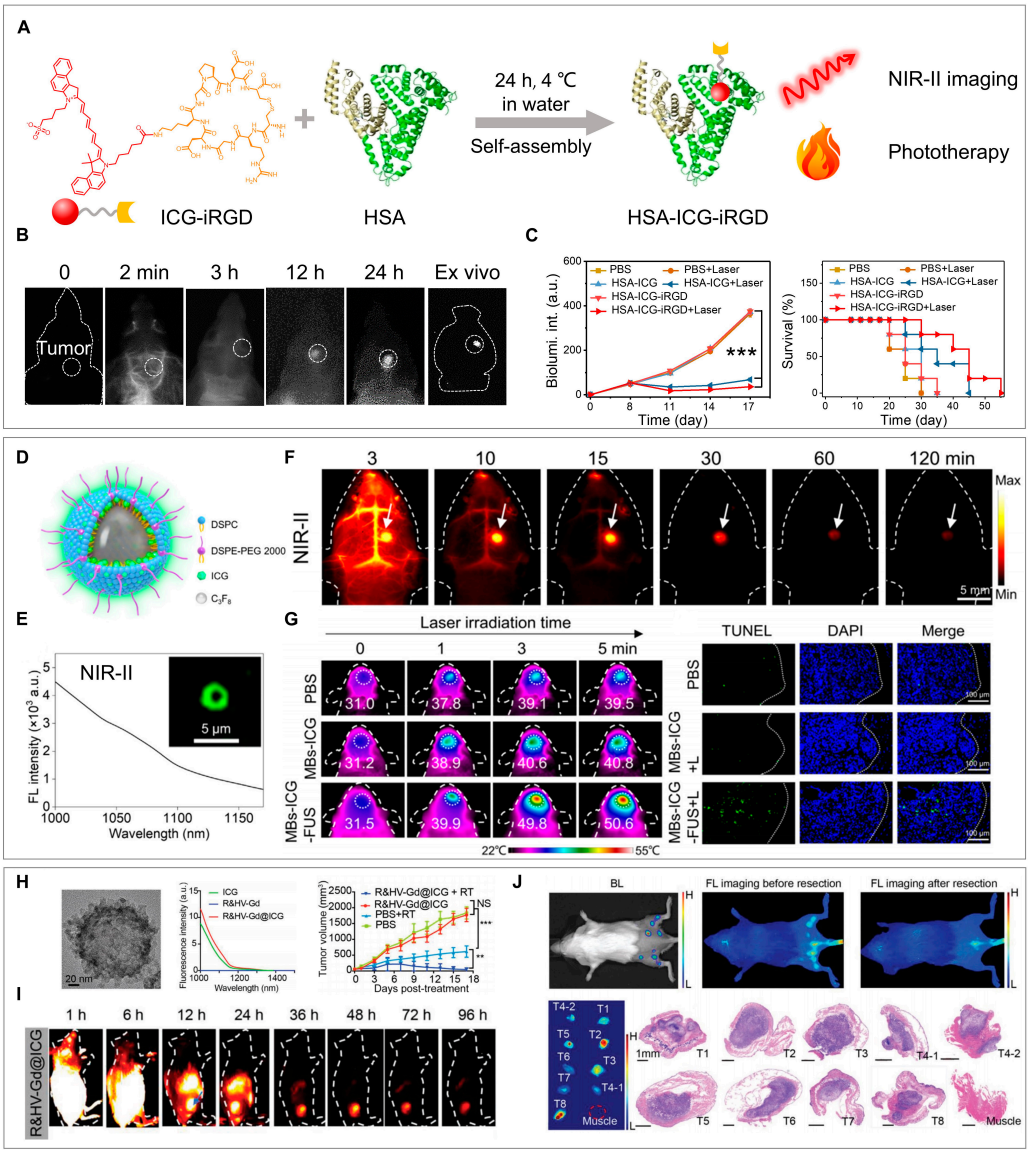
ICG-based probes for NIR-II imaging-guided therapy. (A) Scheme of HSA-ICG-iRGD formation and its application for NIR-II imaging and phototherapy of glioma. Reprinted from [125] with permission from Wiley. (B) In vivo and ex vivo NIR-II imaging of HSA-ICG-iRGD in an orthotropic U251 glioma tumor model. (C) Bioluminescence intensity and survival curves of U251 glioma-bearing mice with different treatments. Reprinted from [125] with permission from Elsevier. (D) Schematic diagram of MBs-ICG. (E) Tailed fluorescence emission spectra in the NIR-II window and fluorescence image of MBs-ICG. (F) NIR-II imaging of MBs-ICG at different time intervals under FUS irradiation. (G) Photothermal images and TUNEL (terminal deoxynucleotidyl transferase–mediated deoxyuridine triphosphate nick end labeling) staining of brain tumors in different treatment groups. Reprinted from [107] with permission from Wiley. (H) High-resolution transmission electron microscopy (TEM), NIR-II emission spectra, and radiosensitization efficacy of R&HV-Gd@ICG in a 4T1 tumor model. (I) NIR-II imaging of R&HV-Gd@ICG in a 4T1 tumor mice model. (J) NIR-II fluorescent image-guided surgically resected 4T1-Luc tumors. Reprinted from [135] with permission from Wiley.

Our group developed small-sized human serum albumin (HSA)–ICG–iRGD nanoprobes to serve as theranostic agents for NIR-II fluorescence imaging and phototherapy of glioma (Fig. 9A to C) [125]. These minute nanoprobes could specifically bind to α_v_β_3_ integrin receptors, which are overexpressed on the tumor cells, by utilizing iRGD peptides. The brain tumors were easily distinguished by NIR-II imaging and eliminated in subcutaneous and orthotopic glioma-bearing nude mice by using PTT. In addition to receptor-mediated targeting, focused US can serve as a physical mechanism to enhance the accumulation of nanomaterials at tumor sites. Our designed ICG-doped microbubbles (MBs-ICG) function as dual-modal contrast agents for NIR-II fluorescence and US imaging. When combined with focused US, they effectively open the blood–brain barrier (BBB) and enhance tumor permeability, enabling high-contrast NIR-II fluorescence imaging of orthotopic gliomas with an SBR of up to 6.2. Moreover, the microbubbles transformed into NPs and deeply penetrated into the brain tumor under focused ultrasound (FUS) irradiation, making it a useful photothermal agent for glioma therapy (Fig. 9D to G) [107]. Although nanocarriers enhance ICG stability to some degree, this challenge persists. Yeroslavsky et al. [126] found that IR-1061 can enhance ICG photostability by reducing singlet oxygen generation through their energy transfer interaction. They encapsulated 2 dyes (IR-1061 and ICG) in polymeric micelles to form NPs for NIR-II fluorescence imaging and cancer therapy. IR-1061 regulates ICG to produce less singlet oxygen and more heat for the phototherapy of MCF-7 cancer cells, thus potentially offering a new method to enhance ICG stability.

Typically, phototherapy is less effective for treating deep-seated tumors. However, a synergistic approach of co-loading ICG and chemical drugs into nanocomplexes has shown to be a promising strategy for the combined chemo-PTT of cancer [127–129]. Su et al. [130] reported a biomimetic liposomal nanomedicine composed of ICG and doxorubicin (DOX) for NIR-II image-guided chemo-phototherapy of pancreatic cancer. The inhibition rate of ICG/DOX-loaded biomimetic liposomes was 91.1%, which was drastically higher than that of other control groups. Recently, for treating gliomas, we engineered apoptotic bodies loaded with DOX and ICG that can cross the BBB through the hitchhiking effect [131]. These findings suggest that a combined photothermal-chemotherapeutic approach was more effective in inhibiting the growth of glioma cells as compared to individual treatments.

Amphiphilic drugs can co-assemble with ICG to form aggregates without the assistance of nanocarriers. This carrier-free strategy maximizes drug loading efficiency, thereby enhancing therapeutic efficacy. Li and colleagues [127] designed a nanodrug by assembly of ICG, methotrexate, and clofarabine (CA) for NIR-II image-guided synergistic photothermal-chemotherapy of cancer. This nanodrug displayed an ultrahigh drug payload (100%) and prolonged blood circulation (>7 d), leading to the complete ablation of 4T1 tumors without recurrence. Additionally, they designed an acid-responsive nanodrug composed of ICG and MTX for the on-demand release of fluorophores and drugs during cancer treatment. This nanodrug served as an NIR-II fluorescence/photoacoustic probe for dual-modal imaging-guided synergistic photothermal-chemotherapy of cervical cancer [132]. By using a similar strategy, Liao et al. [133] constructed nanoflowers named ICG ⊃ EDOX through self-assembly of ICG, DOX, Cu^2+^ ions, and (−)-epigallocatechin-3-gallate (EGCG). The assemblies quenched the fluorescence of ICG and DOX and improved the stability of fluorophores and drugs. Under acidic conditions or laser irradiation, DOX and ICG could be released on-demand with enhanced fluorescence for NIR-II image-guided photothermal-chemotherapy of 4T1 tumors. Developing more strategies for the delivery of both ICG and therapeutic agents, including hydrophobic and hydrophilic drugs, nucleic acids, antibodies, and immune adjuvants, has great potential for the design of new NIR-II imaging-guided theranostic probes.

In addition to the aforementioned organic complexes of ICG, hybrid nanocomplexes composed of ICG and inorganic matrix have been developed for NIR-II imaging-guided cancer theranostics. Huang and colleagues [134] prepared a photochemotherapeutic agent (ICG-labeled Abraxane@MoSe_2_) by combining Abraxane with MoSe_2_ and labeling it with ICG. Under the guidance of NIR-II imaging, the authors achieved synergistic photothermal-chemotherapy of pancreatic tumors through laser irradiation. Subsequently, in 2022, Zhang and colleagues [135] developed a biodegradable nanoprobe named R&HV-Gd@ICG for NIR-II image-guided surgery and enhanced radiosensitization of 4T1 tumors (Fig. 9H to J). This nanoprobe was composed of mesoporous Gd nanospheres, ICG, and iRGD peptides, and it served as an NIR-II fluorescence/MRI dual-modal probe for image-guided surgical resection of residual tumors and improved sensitivity to radiotherapy. Unlike many other nonbiodegradable inorganic nanomaterials, this nanoprobe can be disintegrated and slowly excreted from urine and feces. Despite some progress made by ICG-loaded inorganic hybrids for NIR-II imaging and therapy, their future prospects are not as promising as those of organic carriers because of their higher safety profile. Further efforts to design smaller (<5 nm) and biodegradable inorganic carriers could be an effective solution to address the biosafety concerns associated with inorganic nanoprobes.

The utilization of ICG with various nanocarriers has pioneered cancer theranostics. Organic and hybrid nanocomplexes show promise in imaging and therapy, yet challenges endure. For organics, drug loading and release optimization is key. Inorganics demand biosafety solutions via novel designs. Future work should center on crafting nanocarriers with enhanced targeting, biodegradability, and biocompatibility. Exploring multimodal imaging and combined therapies with ICG probes may enable precise diagnosis and effective treatment of complex diseases. Personalized theranostics based on ICG nanocarriers, considering patient individuality and disease diversity, is also a prospective path. Overall, continuous innovation and interdisciplinary efforts are anticipated to translate preclinical achievements into clinical gains and transform cancer and disease treatment.

## Clinical Applications and Trials

### Clinical applications of ICG as an NIR-II imaging probe

**Fig. 10.**
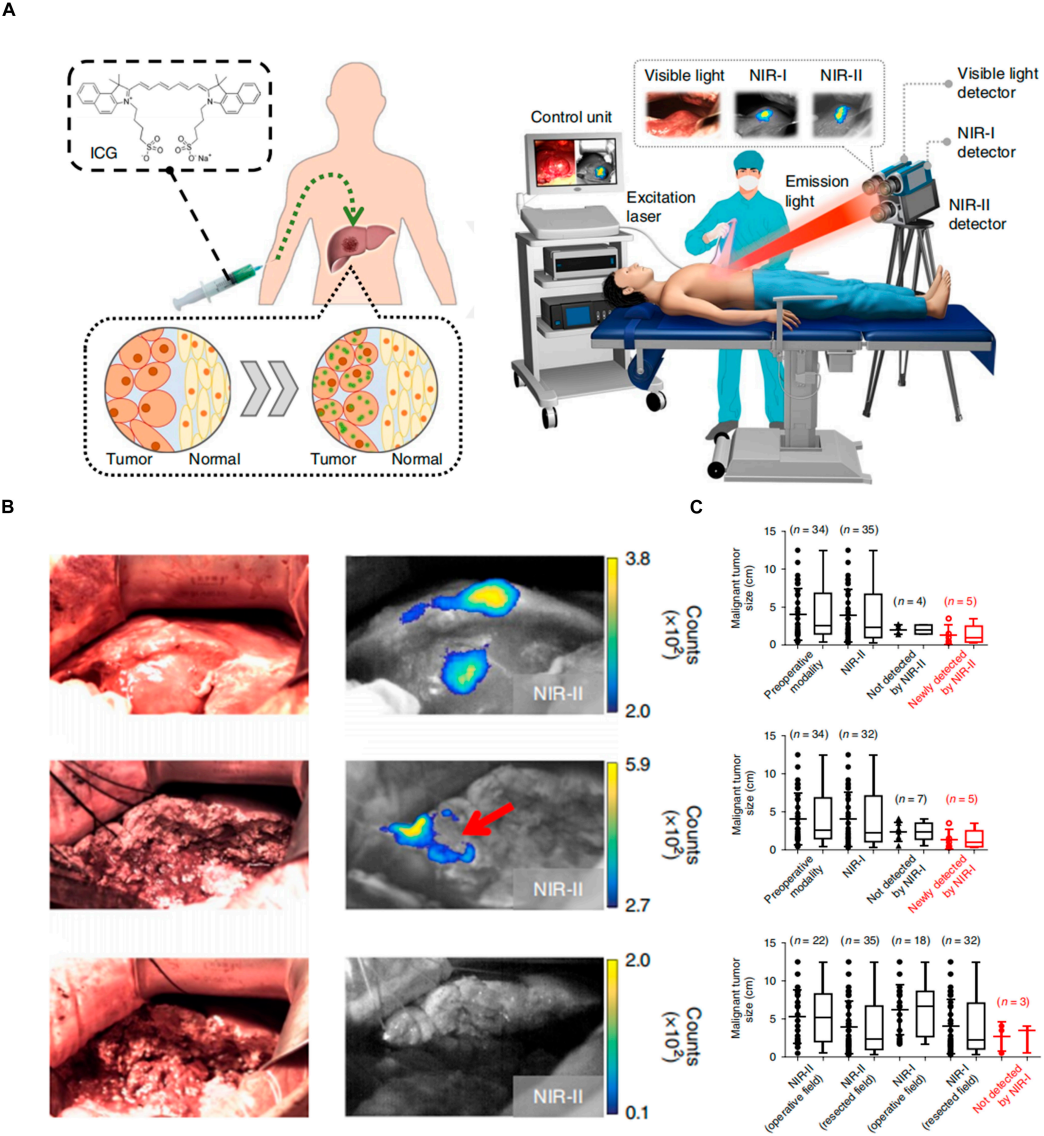
Clinical application of ICG in NIR-II imaging-guided surgery. (A) Schematic diagram of human liver tumor surgery guided by multispectral optical imaging with ICG as probes. Reprinted from [140] with permission from Nature Publishing Group. (B) Intraoperative NIR-II fluorescence imaging-guided tumor surgery. (C) Comparison of the tumor detection ability of NIR-I/II imaging.

In recent years, following preclinical investigations, ICG has gained traction as an NIR-II imaging probe for clinical diagnosis and intraoperative guidance [136–138]. Xu and colleagues [139] conducted a pilot study using ICG to stain extracted human teeth for NIR-I/NIR-II dental imaging and compared it with x-ray imaging. ICG-assisted fluorescence imaging showed unique advantages in visualizing cracked teeth and caries and might serve as a noninvasive method for diagnosing dental diseases in clinics. In 2020, a notable milestone was achieved in the field of medical imaging with the utilization of an NIR-II imaging instrument and ICG for imaging-guided surgeries in patients [140]. Tian et al. successfully conducted fluorescence-guided resection of liver tumors in 23 patients, thereby marking a pivotal advancement. The outcomes indicated that intraoperative NIR-II imaging exhibited a considerably higher tumor detection efficiency (100% versus 90.6%), a better tumor-to-normal ratio (5.33 versus 1.45), and an increased tumor detection rate (56.41% versus 46.15%) as compared to NIR-I imaging (Fig. 10). Additionally, Tian et al. conducted many NIR-II fluorescence-guided surgeries by using ICG as a probe for various clinical applications, including the detection of cervical cancer lesions, identification of pelvic nerves, and detection of bile leaks [141–143].

These findings reinforce the increasing role of advanced imaging modalities for managing complex surgical cases. Cao et al. [144] reported the first investigation of multispectral fluorescence (NIR-I/IIa/IIb) imaging equipment for the precise detection of blood vessels and surgical boundaries. Small capillaries with FWHM as high as 182 μm were observed in NIR-IIb images. The authors also selected 7 patients with grade III/IV glioma and injected them with ICG for surgical resection of glioma, which drastically reduced the blood loss volume of patients. Shi et al. [145] also investigated the clinical benefits of ICG-based NIR-II image-guided surgery for glioma patients. The complete resection rate of the NIR-II imaging group (100%, 15/15 patients) was considerably higher than that of the traditional white light imaging group (50%, 9/18 patients). Moreover, the detection rate, accuracy, and overall survival of the NIR-II image-guided treatment group were enhanced, thus showing the high potential of this technique in clinical applications. Apart from surgical resection of tumors, ICG and NIR-II imaging systems are also applied in microsurgery for the imaging of blood vessels. Cheng and colleagues [146] successfully improved the operational efficiency of 39 patients in microsurgical applications, including vascular anastomosis, finger replantation, and flap transplantation.

### Clinical applications of NIR-II ICG-based probes

In addition to free ICG, a lipiodol-nanoICG formulation utilizing a super-stable homogeneous intermix formulating technology has been developed. This innovative formulation was subsequently used for surgical navigation following long-term embolization treatment. The authors also demonstrated its efficacy in facilitating precise fluorescent laparoscopic resection of hepatocellular carcinoma (HCC) in clinical practice, thus highlighting the potential for enhanced imaging and navigation during complex surgical procedures. This advancement further illustrates the versatility and applicability of ICG-based formulations in improving surgical outcomes [147,148].

Recently, Tian and colleagues [149] reported the use of immunoglobulin G (IgG)–IRDye800CW, which facilitated precise resection of HCC from the cirrhotic liver tissue. The high-contrast NIR-II fluorescence imaging provided clear delineation of margins, which greatly improved surgical precision. The results underscored the feasibility of employing IgG-IRDye800CW for guiding hepatic surgeries while minimizing damage to hepatic function. This advancement introduces a promising new probe for clinical applications, further expanding the toolkit available for surgeons in complex procedures.

Additionally, ICG–protein conjugates are also being used in the clinic. Zhang’s group incubated the Indocyanine Green–Sacituzumab Govitecan-hziy (ICG-SG) probe with excised breast tissue, followed by NIR-II fluorescence imaging to identify tumor regions and delineate tumor boundaries. The accuracy of the fluorescence imaging was validated through pathological diagnosis. This method aims to provide a new probe for distinguishing between benign and malignant breast tissue [150].

In clinical applications, ICG and ICG-based probes as NIR-II fluorophore agents have proven effective for fluorescence imaging-guided surgeries across a range of conditions, including liver cancer, gliomas, giant mediastinal tumors, gastric cancer [151,152], and blood vessel imaging [153] These studies not only confirm the accuracy and safety of NIR-II fluorescence-guided surgical techniques but also highlight the crucial role of ICG and ICG-based probes in enhancing intraoperative decision-making and benefiting patients.

### Clinical trials of NIR-II ICG-based probes

In addition to these published articles, there are currently over 100 ongoing clinical trials based on ICG fluorescence probes. This paper primarily focuses on clinical trials involving ICG-based complexes for NIR-II imaging. Recently, Y. Ning from the Second Hospital of Shanxi Medical University conducted a prospective study for early detection of peripheral artery disease by the imaging performance of ICG in the NIR-II window (NCT06565819). The study recruited patients diagnosed with type 2 diabetes without peripheral artery disease from the Department of Endocrinology at the Second Hospital of Shanxi Medical University. Following the injection of ICG, NIR-II imaging was performed to record the timing and corresponding fluorescence intensity, and patients underwent follow-up evaluations at 6 months and 1 year after imaging. The effectiveness of NIR-II imaging for the early diagnosis of peripheral artery disease was assessed using Doppler US (DUS) results as the reference standard. Besides the application of ICG, several ICG–protein conjugates are also being tested in clinical trials. A clinical trial conducted by Hu Zhenhua aims to verify the feasibility of using an NIR-II endoscope and a VEGF-A targeting fluorescent probe to image abnormal tissues in the gastrointestinal tract (NCT06430372). The results of these clinical trials will further expand the applications of ICG, advancing the clinical translation of ICG-based complexes and ultimately benefiting patients.

## Conclusion and Prospects

In summary, ICG has garnered renewed interest due to its strong tail emission characteristics in the NIR-II biological window. Numerous studies have focused on developing ICG-based probes to overcome its inherent limitations, such as photo-instability, short half-life, and lack of molecular targeting capability. ICG-based probes, including ICG derivatives, ICG-conjugated molecular probes, ICG–antibody conjugates, and ICG–nanocarrier complexes, play a crucial role in NIR-II imaging-guided disease diagnosis and treatment, particularly in oncology, where notable progress has been made in the precise diagnosis and treatment monitoring of conditions such as gliomas, CRC, and breast cancer. Additionally, there are exploratory applications of ICG in other fields, including cardiovascular diseases and dental conditions. Nonetheless, numerous obstacles remain to be overcome before ICG-based probes can be broadly utilized in clinical settings.

1. Clinical translation of ICG-based probes

Although ICG is a biologically safe molecule, ICG-based probes still pose risk of high organ accumulation and potential long-term toxicity in vivo. Thus, all newly developed ICG-based probes need to be strictly tested for clinical translation. To accelerate the translation process, the following measures should be implemented: (a) Carriers: the nanocarriers should have good biocompatibility, and clinically approved vehicles should be preferred when designing ICG-loaded nanocomplexes to reduce potential toxicity risks. (b) Metabolism: Develop probes that can be efficiently metabolized or biodegradable in vivo, ensuring that degradation products are safe and can be metabolized. (c) Appropriate application scenarios: Intravenous injections often pose safety concerns, so selecting suitable application scenarios can aid in the clinical translation of specific ICG-based probes. For instance, probes can be utilized for pathological identification of excised tissues without entering the body, or during lymph node dissections, where probes can be removed after entering the lymph nodes. (d) Multifaceted participation: Involve government, professional organizations, and marketing agencies in the clinical trials of new imaging agents to address the lack of experience among researchers.

2. Imaging systems and algorithms

In recent years, various NIR-II imaging systems have emerged, effectively meeting the needs of both research and clinical applications. However, there remains considerable room for improvement in these systems: (a) High-sensitivity, low-cost detectors: Developing cost-effective and highly sensitive NIR-II imaging detectors is crucial for promoting the widespread application of NIR-II imaging. (b) Excitation light sources: A stable and uniform excitation light source is a key factor in obtaining high-quality images. Using multi-angle illumination helps achieve uniform light density distribution in the target area, greatly enhancing imaging quality. Recently, low-energy light-emitting diode lights have been explored as excitation sources, which can help mitigate the photobleaching effects on ICG-based probes. (c) Imaging algorithms: With the continuous advancement of artificial intelligence technologies, training deep neural networks for the automated processing and analysis of NIR-II fluorescence imaging data holds promise for further expanding the application domains of probes and instruments, thereby improving their efficacy. (d) Portability: Miniaturized and portable imaging devices are better suited for use in complex clinical settings, thereby better addressing actual medical needs.

3. New dimensions in ICG-based probe imaging

Fluorescence imaging in the NIR-II region is becoming increasingly important for tumor diagnosis. While traditional fluorescence intensity imaging has been crucial, its accuracy can be limited by various factors, including imaging system parameters, tissue depth, and the amount of dye absorbed by the tumor. In contrast, fluorescence lifetime imaging, which relies on photophysical properties, is less affected by these variables and introduces new possibilities in fluorescence imaging. Notably, fluorescence lifetime imaging using ICG-based probes demonstrates greater precision and improved SBR when delineating tumor boundaries. As imaging technologies and probes continue to advance, the application of NIR-II fluorescence lifetime imaging—tailored to the physiological environment and targets of lesions—will substantially enhance the development of high-resolution and high-sensitivity precision diagnostics and therapeutics.
